# Technological Potential of a Lupin Protein Concentrate as a Nutraceutical Delivery System in Baked Cookies

**DOI:** 10.3390/foods10081929

**Published:** 2021-08-19

**Authors:** Joana Mota, Ana Lima, Ricardo B. Ferreira, Anabela Raymundo

**Affiliations:** 1 LEAF, Instituto Superior de Agronomia, Universidade de Lisboa, 1349-017 Lisbon, Portugal; agusmaolima@gmail.com (A.L.); rbferreira@isa.ulisboa.pt (R.B.F.); anabraymundo@isa.ulisboa.pt (A.R.); 2 Faculty of Veterinary Medicine, Universidade Lusófona de Humanidades e Tecnologias, Campo Grande, 376, 1749-024 Lisbon, Portugal

**Keywords:** lupin extract, anti-MMP activity, cookies, physicochemical properties, HT29, MMP-9

## Abstract

Previous reports have shown that lupin protein extracts (LE) contain a polypeptide named deflamin with a potent matrix metalloproteinase (MMP)-9 inhibitory activity. The aim of our study was to develop an efficient delivery method for incorporating deflamin into cookies using different alternative flours. A lupin protein concentrate (10 g protein/100 g cookie dough) was added to gluten and gluten-free flours to produce savoury cookies, and its impacts on the physical properties of doughs and cookies, as well on the maintenance of deflamin’s anti-MMP-9 activity, were analysed. The results showed that the biochemical compositions of all cookies with LE presented higher protein and ash contents when compared to the control cookies. Rice, buckwheat and oat doughs were firmer than the others, whereas the addition of LE to kamut and buckwheat flours made cookies significantly firmer than the controls. Additionally, strong interactions between LE and several flours were observed, yielding different impacts on the MMP-9 bioactivity. Overall, the only flour that did not interfere with the desired nutraceutical activities was buckwheat, with 60% MMP-9 inhibitory activity and a concomitant reduction of colon cancer migration; hence, buckwheat flour was revealed to be a good vehicle to deliver bioactive deflamin, showing strong potential as a functional food to be used in preventive or curative approaches to gastrointestinal diseases.

## 1. Introduction

Consumers’ rising concerns regarding health and well-being have resulted in increasing attention being given to bioactive food compounds that exhibit health-promoting effects. In fact, the diet-based prevention of chronic diseases, such as inflammation and cancer [1,2,3], has become a great focus of attention, based on the principle that preventing a disease is more effective than curing it [1,2,3]. In particular, the enhancement of the nutritional and health properties of food products through the incorporation of nutraceuticals has become a topic of great interest to both consumers and the food industry [1]; however, one of the great constraints related to the direct incorporation of nutraceuticals and bioactive compounds into food products is in identifying adequate materials and production techniques [3]. Indeed, the choice of the correct food matrixes is a key step, since it may have a strong impact on the bioactivity. Baked snacks such as crackers and biscuits are usually well accepted and consumed throughout the world, and can be excellent vehicles for nutraceutical and protein enrichment because of their wide consumption and long shelf life [4]. Previous studies from our group have incorporated a GRAS-safe lupin protein concentrate in sweet baked cookies, using gluten and gluten-free flours, which increased their nutrition potential [5]. Lupin seeds are well known to provide a diversity of essential nutrients, including carbohydrates, dietary fibre, protein, minerals and vitamins [6], which exhibit important biological activities, such as lower the glycaemic index and decreasing hypertension and cholesterol [6]. Amongst legumes, lupin has the highest protein content [7], providing it great potential for consumption. Furthermore, Lima et al. [8] showed that the protein extract from lupin can reduce cell migration in colon carcinoma cells through matrix metalloproteinase (MMP)-9 inhibition [8], mostly due to the presence of a protein oligomer nutraceutical named deflamin, which seems to be an excellent MMP-9 inhibitor [9]. Isolated deflamin also reduced inflammation and the expression of inflammation mediators in colitis models in vivo [9]. From this point of view, the incorporation and maintenance of the nutraceutical activities of lupin in baked cookies could be used to produce functional foods for the prevention and amelioration of chronic diseases related to MMP-9, such as inflammatory bowel diseases and colon cancer [10,11]. The incorporation of a lupin protein extract instead of lupin flour also presents several advantages, including the elimination of potentially toxic compounds, such as digestive enzyme inhibitors and antinutritional compounds [12], guaranteeing the presence of sufficient amounts of active deflamin [9]. Nonetheless, previous results have demonstrated that the MMP-9 inhibitory activity of deflamin was reduced in the presence of sugar-containing dough; therefore, in the present study we tested whether the lupin protein concentrate could be used as a vehicle for the MMP-9-inhibitory compound deflamin in savoury baked cookies. The use of wheat flour substitutes as acceptable alternatives reduces calorie intake and increases the availability of healthier snacks on the market, in turn reducing inflammation [13,14]; therefore, we tested the incorporation of such flour substitutes in different types of matrices, such as gluten-containing flours from spelt and kamut, which have been reported to cause less inflammatory responses than *Triticum aestivum* [15], oat, rice and buckwheat flours, which are the most consumed gluten-free flours [16].

Overall, our main goal was to assess the impacts of the addition of lupin protein extract on the physicochemical properties and bioactivities of savoury cookies with and without gluten flours from different origins. Our main goal was to develop a staple food that could act as a vehicle for the ingestion of a deflamin-rich lupin concentrate in the form of baked cookies, without changing its status as a functional food, whilst implementing adaptation strategies to reduce the impacts of inflammatory and cancer diseases through cost-efficient and applicable dietary approaches.

## 2. Materials and Methods

### 2.1. Preparation of Lupin Seed Protein Extract

*Lupinus albus* seeds were obtained from Jouffray Drillaud, France. Protein extraction was performed according to Mota et al. [5] with slight alterations, using approximately 100 g ± 0.1 g of powdered *L. albus* seeds with 1:10 (*w*/*v*) milli-Q water. The extract was stirred overnight at 4 °C and was filtered using Miracloth. The homogenate was maintained in a 100 °C water bath for 30 min and then filtered again. The extract was kept frozen at −80 °C and lyophilized (Edwards, Crawley, UK). The final quantity obtained was 50 g of lupin extract (LE) [8].

### 2.2. Flours Composition

The nutritional compositions of the gluten-containing flours, kamut and spelt; of the gluten-free flours buckwheat, rice and oat; and of the remaining ingredients, namely sunflower oil, salt and baking powder, were obtained as described by Mota et al. [5].

### 2.3. Cookies Preparation

Savoury cookies were prepared using 65.5% flour, 1% salt, 1.5% baking powder, 7.5% sunflower oil and 24.5% water, according to a previously optimized model formulation [17]. In all tested samples, the ingredients were added in similar quantities, except for the flour, which in LE cookies was substituted by 10% LE, in contrast to controls, to which no LE was added. The quantity of LE contained in the cookies was established using previous data [5]. All ingredients were mixed in a food processor (Bimby, Vorwerk), where 100 g batches were mixed for 15 s at speed 4. The savoury cookies were moulded, baked and stored as described by Mota el at [5]. On the other hand, the sweet cookies used in this study were prepared according to Mota et al. [5], using 54% flour, 15% sugar, 18% margarine, 12% water and 1% baking powder.

### 2.4. Dough Rheology

Rheological measurements were performed according to Mota et al. [5], using a controlled stress rheometer (Haake MarsIII—Thermo Scientific, Karlsruhe, Germany) with a UTC–Peltier system. Frequency sweep tests were performed within the viscoelastic linear region, which was previously defined through a stress sweep test, at 1 Hz using a serrated parallel-plate geometry with a 20 mm diameter. Dough pieces were compacted with a 1.5 mm gap and the edge parts were coated with liquid paraffin to prevent moisture losses during tests. The temperature used for the stress and frequency sweeps was 25 °C.

### 2.5. Colour Analysis

The colour of the cookies samples was measured using a Minolta CR-400 (Japan) colorimeter. The method used was previously described by Mota et al. [5]. The measurements were performed under the same light conditions using a white standard (L* = 94.61, a* = −0.53, and b* = 3.62) at control temperature, replicated ten times for each sample (control and LE) 24 h after baking.

Total colour differences between control and LE cookies were assessed using Equation (1):

ΔE* = (ΔL*·2 + Δa*·2 + Δb*·2 ) ½(1)

### 2.6. Texture Analysis

Texture analysis was performed in a TA.Xtplus (StableMicro Systems, Godalming, UK) texturometer. The measurements were performed at 20 °C  ±  1 °C, in a temperature-controlled room.

#### 2.6.1. Dough Texture

For the dough texture profile analyses (TPA), samples were subjected a simulation of the action of double chewing using the two-bite test, according to the method previously described by Mota et al. [5]. Each analysis was repeated eight times.

#### 2.6.2. Cookie Texture

Each cookie’s texture was assessed with a penetration test as described earlier by Mota et al. [5]. These tests were reproduced at least eight times for each cookie (control and LE) 24 h after baking.

### 2.7. Water Activity Determination

The water activity (a_w_) was established using a thermohygrometer (HygroPalm HP23-AW, Rotronic AG, Bassersdorf, Switzerland) at 20 °C ± 3 °C. After 24 h of baking, the tests were performed by crushing the cookies, with each cookie (control and LE) measured in triplicate.

### 2.8. Chemical Compositions of the Cookies

The approximate chemical compositions of the cookies were evaluated based on powdered samples. According to the AOAC 950.36 official method for baked products, the protein content was evaluated using the Kjeldahl method. The total nitrogen content was multiplied by 5.7 (conversion factor) to acquire the cookie crude protein according to Batista et al. [17]. Crude fat was measured using ether extraction according to AOAC 2003.05. A minimum of 1.5 g of each cookie (with and without LE) was weighed into a 26 mm × 60 mm cellulose extraction thimble. The content of petroleum ether lipids was evaluated by Soxtec extraction (Soxtec System HT 1043/1046 extraction unit (Tecator AB, Höganäs, Sweden), with 15 min of boiling and 60 min of rinsing, followed by 15 min of drying. Finally, the lipid content was determined gravimetrically. Ash content, representing the inorganic fraction of the cookies, was measured by incineration at 550 °C in a muffle (AACC 08-01.01). Moisture content was determined according Mota et al. [5]. Total carbohydrates were calculated by difference.

### 2.9. Bioactivities of the Cookies

#### 2.9.1. Protein Extraction

Approximately 10 g ± 0.1 g of cookie was milled in a coffee grinder (Taurus Aromatic, Oliana, Spain) and the resulting cookie powder was resuspended in 40 mL of 100 mM Tris–HCl buffer (pH 7.5, 1:4 *w*/*v*) and stirred for 4 h at 4 °C. Each sample was then centrifuged at 12,000× *g* for 30 min at 4 °C, then supernatants were collected and stored at −20 °C.

#### 2.9.2. Specific MMP-9 Inhibition

To determine the MMP-9 inhibitory activity levels of different cookies, the samples were prepared as described in Section 2.9.1. The supernatants were stored at −20 °C. MMP-9 inhibition was tested using the DQ fluorogenic assay as previously described [8]. To compare the MMP-9 inhibitory activity levels of savoury cookies versus sweet cookies, we produced sweet cookies using the same flours, using the method already described by Mota et al. [5].

#### 2.9.3. Wound Healing Assay

The human colon adenocarcinoma cell line HT29 (ECACC 85061109), obtained from a 44-year-old Caucasian female, was used in this study. HT29 cells were maintained as described by Lima et al. [8]. For cell migration evaluation, the wound healing assay was performed as described by Lima et al. [8]. In this test, a cell-free area is established in a confluent monolayer by eliminating the cells using a scratch. Then, the cells tend to migrate into the gap. The measurements of the migrated area are determined at 0 and 48 h. Each well contained cookie protein extract at a concentration of 100 μg.mL^−1^ of total soluble protein.

### 2.10. Statistical Analyses

The experimental data were acquired in triplicate and were analysed using SigmaPlot (version 12.5), as described previously [5]. Analysis of variance (one-way ANOVA) was used to calculate the differences between samples *p* < 0.05 representing significance levels of 95% and *p* < 0.001 of 99.99%. Differences between treatments were compared using Tukey’s test.

## 3. Results

With the emergence of the nutraceutical industry, the development of novel candidates with high applicability, as well as being innovate and easy to manufacture industrially, has become a major goal; however, the delivery of such nutraceuticals in functional foods is often challenging. The food matrix is the first and one of the most important factors affecting the biological fate of a nutraceutical. The delivery systems should be congruent with the food matrix and should not influence the properties of the food product (in terms of aroma, taste, appearance and texture) [3]. For deflamin, its resistance to heat and its ability to be easily concentrated in the form of a lyophilized lupin protein powder extract meant that it could be easily introduced in baked cookies [18]. Nonetheless, our previous preliminary results suggested that the type of flour, and particularly the presence of sugar, had strong impacts on deflamin’s activity. 

As such, the propose of the present work was to deliver the GRAS lupin protein concentrate previously developed in [5] in savoury baked cookies, aiming to maintain its effective anti-MMP-9 functional activities. The lupin extract (LE) was, therefore, combined with different types of flours, with and without gluten, including spelt, kamut, oat, rice and buckwheat, with no addition of sugar.

### 3.1. Physical Characteristics of the Cookie Dough

We first set out to determine the impacts of the addition of LE on the technological properties of the different doughs. The parameters obtained through TPA, such as the firmness, adhesiveness and cohesiveness, were the texture properties used to compare doughs, since they can distinguish variations between samples [19].

**Figure 1:**
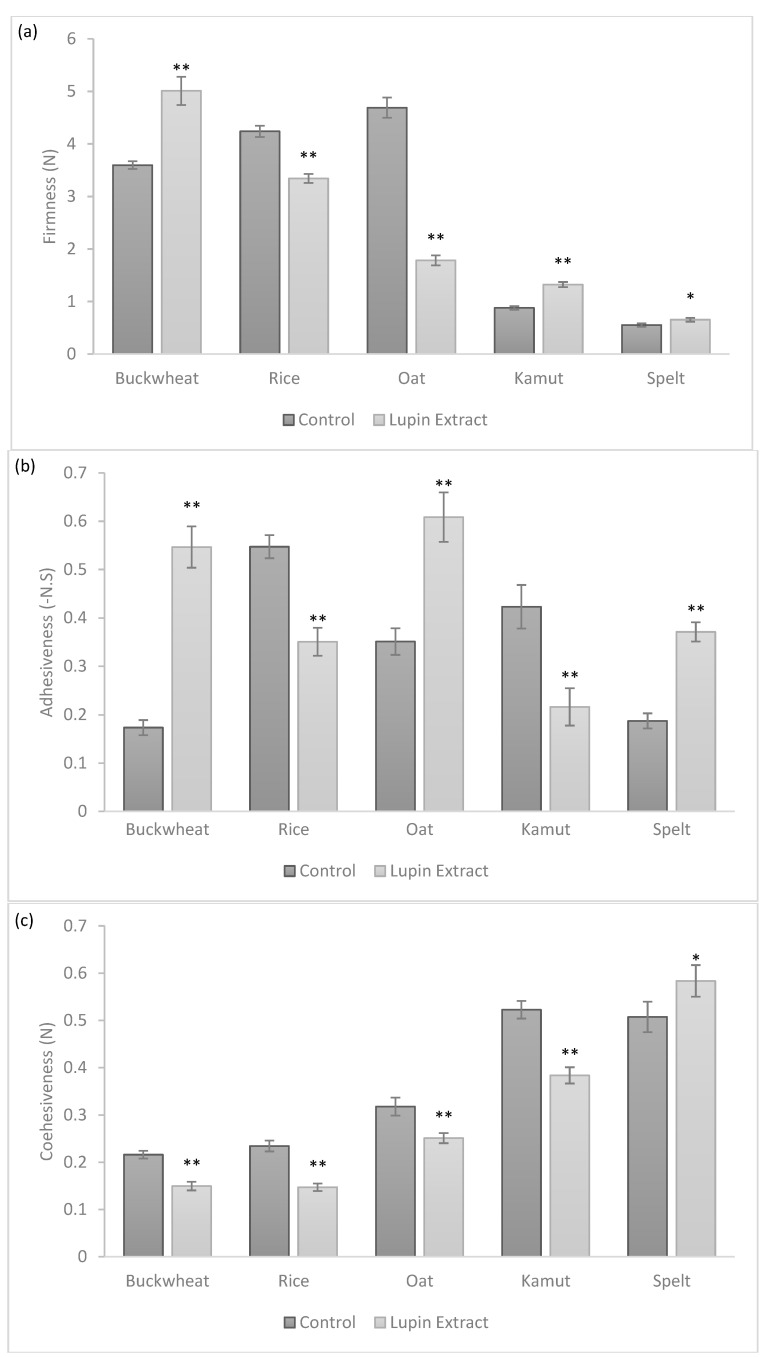
Texture parameters of control and LE cookies containing alternative flours (buckwheat, rice, oat, kamut and spelt): (**a**) firmness; (**b**) adhesiveness; (**c**) cohesiveness. Note: * represents *p* < 0.05 and ** represents *p* < 0.001 when compared with control cookie.

Figure 1 shows the firmness (a), adhesiveness (b) and cohesiveness (c) values for control and 10% (*w*/*w*) LE doughs produced with five alternative flours. Compared to the control dough, the ancient grain doughs (kamut and spelt) were less firm and more cohesive, while the oat dough was firmer and less adhesive than the other doughs. These results are in agreement with Angiolini and Colar [20], who conducted a study on bread enriched with oat, buckwheat, spelt and kamut. The presence of the gluten protein matrix increases the air retention capacity of the structure [21], causing less firm doughs.

The impact of the LE on the cookies is in agreement with the results obtained by Mota et al. [5] for the incorporation of LE in sweet cookies. All parameters tested showed significant differences (*p* < 0.05) between LE and control cookies. In terms of firmness, the gluten-free flours, namely buckwheat and rice flours with LE, showed values of 5 N and 3.3 N, respectively, being firmer when compared to the other alternative flours. The more adhesive doughs were those prepared from oat and buckwheat flours at 0.55 N and 0.6 N, respectively; however, regarding cohesiveness, the behaviour was opposite in comparison with firmness, because the LE cookie with gluten-free flours were less cohesive and the ancient grain flours were more cohesive. Clearly, as expected, the gluten proteins played a crucial role in the matrixes of the cookies, as was reported in other studies [22,23]. On the other hand, the distinct behaviours observed amongst gluten-free flours likely resulted from: (i) different extensions of the interactions between LE, proteins and polysaccharides from the gluten-free starch flour; (ii) the different hydration level of each flour. In fact, Boucheham et al. [24] demonstrated that legume seed flours have higher water absorption capacity (WHC) levels than cereal flours, resulting from the higher protein contents present in legume-derived ingredients. In fact, protein–water interactions are related to the WHC [25], meaning the addition of LE should influence the hydration levels of flours and cause less cohesiveness in the corresponding doughs.

**Figure 2:**
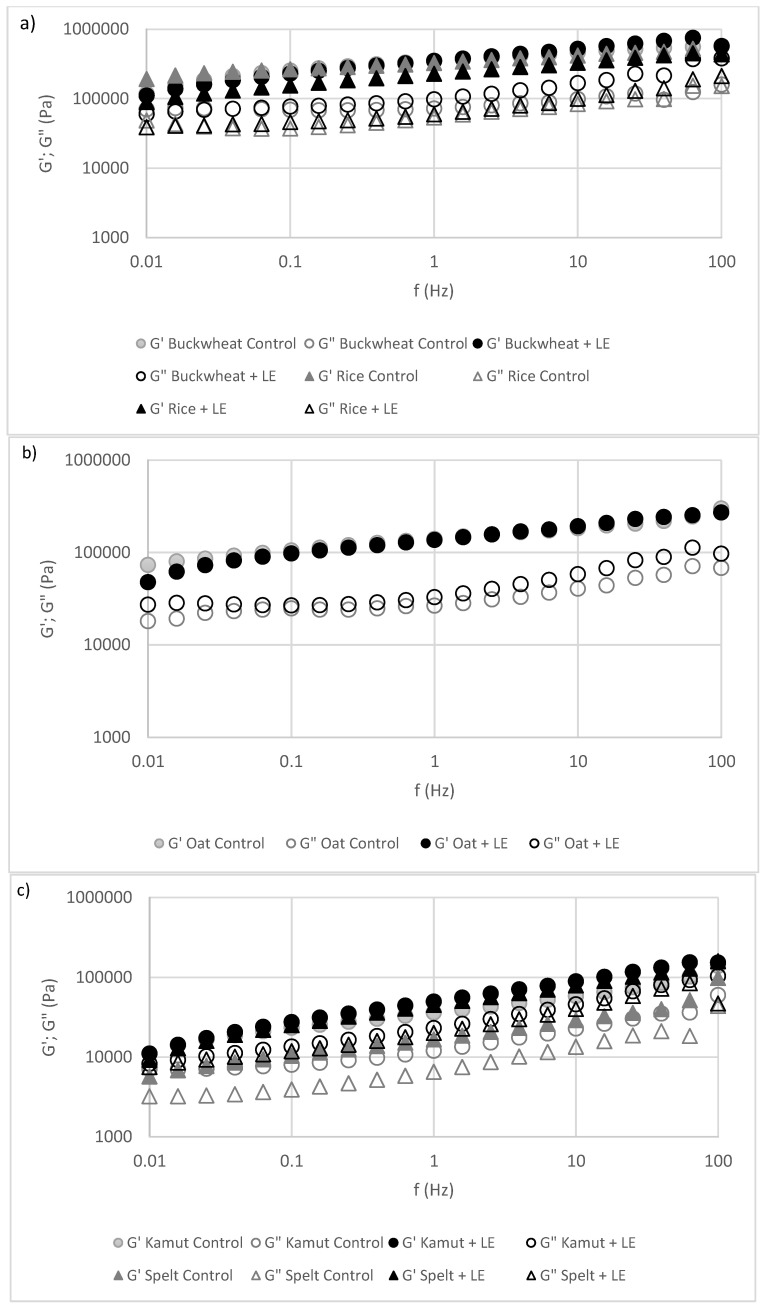
Mechanical spectra of control and LE doughs prepared with alternative flours: (**a**) gluten-free flours, buckwheat and rice; (**b**) oat; (**c**) gluten flours, kamut and spelt. Close symbols represent G’ (elastic modulus) and open symbols represent G” (viscous modulus).

In Figure 2, the viscoelastic behaviours of the five alternative flours in cookie doughs with and without LE are represented.

The G’ (storage modulus) and G’’ (loss modulus) increased with increasing frequency range and revealed a weak gel-like rheological behaviour that is characteristic of cookie doughs and in agreement with the results demonstrated by Mota et al. [5] and Raymundo, Fradinho and Nunes [26]. Regardless of the nature of flour, the mechanical spectra were similar among samples with no drastic changes; consequently, the incorporation of LE did not induce changes in the structures of the doughs. This trend was not observed for spelt flour, since the G’ and G’’ values for the LE-containing dough increased when compared to the respective control dough. This behaviour, i.e., the increase of the viscoelastic functions for spelt flour as the result of the addition of LE, likely resulted from the higher content of free sugars presented in this flour. The presence of a high sugar content has a huge impact on the water absorption [27] and consequently on the development of the structure development between LE proteins and spelt proteins and polysaccharides. Similar behaviour was also noted by Sahagún and Gómez [28] after introducing 30% potato protein into corn cookie doughs and by Mota el al. [5] after the incorporation of 10% lupin protein into buckwheat flour to produce sweet cookies.

### 3.2. Physical Properties of Cookies

**Figure 3:**
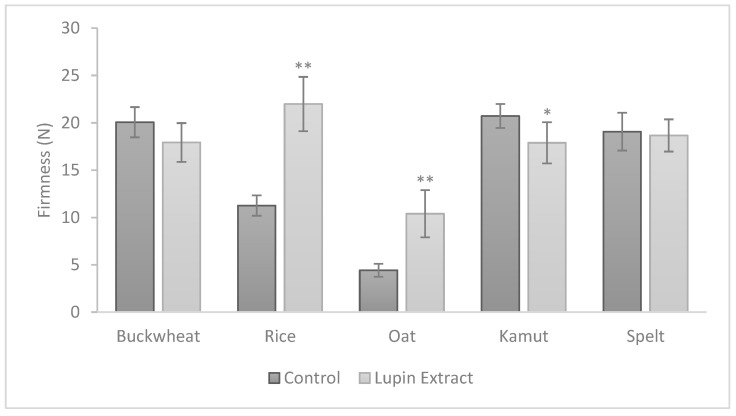
Firmness levels of control and LE cookies 24 h after baking with five different flours. Values are the averages of six experiments ± SD. Note: * represents *p* < 0.05 and ** represents *p* < 0.001 when compared with the corresponding control cookie.

The physical properties of cookies frequently determine their attractiveness and desirability (or undesirability). As such, firmness (Figure 3), the colour parameters (Table 1) and water activity levels (Table 1) of all cookies with and without lupin extract were evaluated.

The texture results of cookies with 10% (*w*/*w*) LE incorporation are presented in Figure 3. The variations observed for several of the studied doughs are reflected compared to the baked cookies, clearly indicating differences in firmness. Cookies prepared with oat and rice flours (without LE) revealed less firmness in comparison to other flours. The impacts of LE were relevant in kamut flour (*p* < 0.05) and very significant in rice and oat flours (*p* < 0.001). Cookies with LE and rice flour were firmer (22 N), whereas cookies with LE and oat flour were less firm (10.4 N). These distinct behaviours are likely related to the macromolecule structures of the five flours due to the absence or presence of gluten. The impacts of LE caused a structural rearrangement leading to diverse interactions amongst these macromolecules (starch and protein interactions) and altered by the heat treatment that exists during cooking. Furthermore, Raymundo, Fradinho and Nunes [26] showed the same behaviour differences between the doughs and the respective cookies.

**Figure 4:**
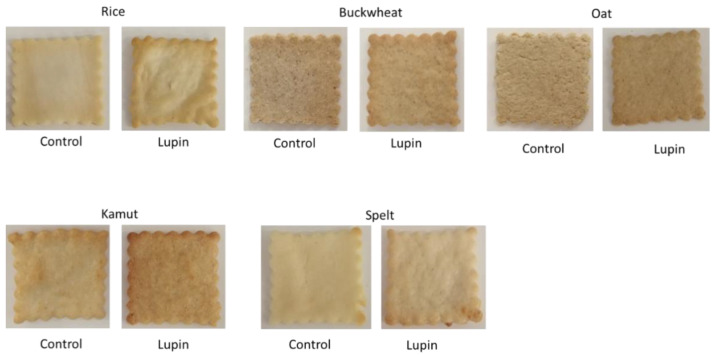
Representative images of control and LE cookies 24 h after baking with five alternative flours.

The impacts of LE on cookie colour are summarized in Figure 4 and Table 1. The ΔE* values were determined to compare the colour variations of cookies with and without LE. In the same table, colour parameters (L*, a*, and b*) and water activity (a_w_) levels for all cookies are shown as well.

The colour variations between the control and LE-enriched cookies expressed in terms of ΔE* values were greater than 5 for all flours, except for kamut, indicating that the colour differences amongst the LE cookies and the controls were visually distinguishable by the human eye [29]. The golden-brown colour resulted from reductions in the lightness parameter (L*) in all LE-containing cookies. Nevertheless, the highest ΔE* values were obtained for rice and spelt flours at 17.13 and 14.64, respectively. These results were likely due to the Maillard reaction, as amino acids and reducing sugars start a flow of reactions throughout heating (higher than 100 °C), generating darker colours [30]; additionally, these two flours in particular were whiter than the others. Despite being darker, the LE cookies presented very appealing colours and their appearance did not suffer negatively, as supported by similar studies [5,26].

The a_w_ values for LE cookies were significantly higher (*p* < 0.001) than those for the control cookies. Moreover, the incorporation of LE increased the a_w_ values for all flours tested, except for spelt flour, which was in agreement with the results shown by Mota et al. [5]. Furthermore, several studies observed increases in a_w_ values with the addition of apple fibre to cookies [31] and with the addition of microalgae with a high protein content [32]. For the lupin-enriched cookies, the increases in the protein content led to lower water-holding capacity values when compared to the respective control flours, supporting the higher a_w_ values. Furthermore, the a_w_ values remained at low levels and the addition of LE did not modify the preservation characteristics of the food products.

### 3.3. Chemical Composition of Cookies

The proximate analysis of foods involves the determination of the principal components, namely ash (total minerals), lipids, protein, moisture and carbohydrates. Table 2 presents the approximate chemical compositions of the cookies prepared with lupin extract.

Lupin-enriched cookie samples exhibited significantly higher (*p* < 0.001) protein contents than the control (Table 2), as expected. LE cookies with buckwheat (13.85%) and kamut (14.53%) flours had the highest crude protein values, while the lowest value was verified in rice flour (10.18%). The enrichment of snacks and biscuits with different protein sources has received extensive attention from several authors; this is the case for the incorporation of microalgae, with studies revealing increases in protein content [32,33]. On the other hand, the ash contents were significantly higher (*p* < 0.05) for rice and kamut flours, with very significant values (*p* < 0.001) for buckwheat, oat and spelt flours when comparing LE cookies with the respective controls. Alomari and Abdul-Hussain [34] and Bilgiçli and Levent [35] demonstrated the same increases in ash content in bread supplemented with different concentrations of lupin flour and with incorporation of lupin flour in wheat cookies, respectively.

Overall, the increases in the amounts of protein and in several physicochemical parameters corroborate that the addition of LE can increase the quality of the savoury biscuits produced. The use of lupin may provide more advantages since this species is exceptional amongst legumes, having some of the highest quantities of digestible plant protein (38%) and dietary fiber (30%) and a particular low quantity of antinutritional compounds, meaning it does not need to be soaked or cooked [33]. Additionally, since sweet lupin consumption is known to decrease blood pressure, improve blood lipids and insulin sensitivity, and favourably alter the gut microbiome, there is increasing interest in this legume as an ingredient to improve the nutritional value of baked goods (particularly gluten-free products) [36,37].

### 3.4. Anti-MMP-9 Activities of Cookies

**Figure 5:**
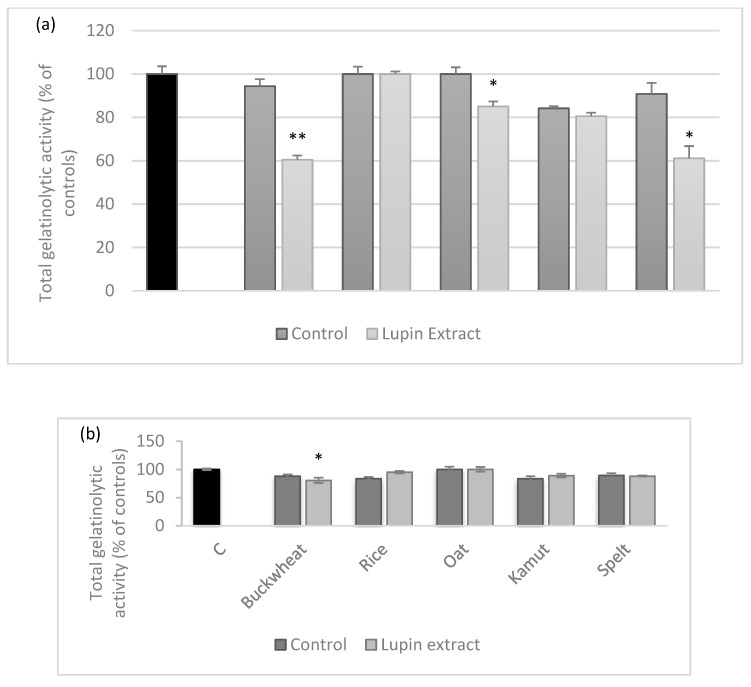
The effects of five different flours with and without LE on MMP-9 activity: (**a**) savoury cookies; (**b**) sweet cookies. The positive control (C) does not inhibit MMP-9, resulting in 100% proteolytic activity. All samples were added at the same volume (80 µL) and gelatinolytic activity was measured. Gelatinase activities are expressed as relative fluorescence as percentages of controls (C) and represent the means of three replicate experiments (*n* = 3) ± SD. * *p* < 0.05 and ** *p* < 0.001 when compared to cookie controls.

Since the main goal of the present work was the use of LE as a delivery system for the previously detected nutraceutical MMPI properties of *L. albus,* we tested the inhibitory effects of all cookies against MMP-9 activity using the standard DQ gelatin assay (Figure 5).

Although the MMPI activity had already been shown to resist baking, the inhibitory activity against MMP-9 was very significantly altered in the presence of the different flours. Figure 5a shows that although all lupin-enriched savoury cookies, except for oat flour cookies, were able to significantly inhibit the gelatinase proteolytic activity (*p* < 0.05) at low levels (around 20%), the only physiologically relevant inhibition levels were observed for buckwheat and kamut flours at 39.6% and 38.9%, respectively, although both flours have been reported as having anti-inflammatory effects. Valli et al. [15] performed an in vitro study in HepG2 cells and reported that kamut bread had an anti-inflammatory activity when compared to other grains, while the anti-inflammatory effects of buckwheat have been reported in in vivo models of intestinal inflammation by reducing colonic mucosa inflammation [38] in comparison with the controls, showing that the activity detected was not due to their intrinsic activity but rather due to the effect of incorporating the LE with anti-MMP-9 activity, as previously reported by Lima et al. [8]. The reasons for the differences found in the different flours could be somewhat related to the amounts of starches and sugary molecules in each one. Indeed, MS sequencing of the oligomer that forms deflamin showed the presence of fragments of beta conglutin, which is well known for its lectin activities. It is feasible that by binding to different sugars in different flours, the deflamin in the LE may lose some of its activity. This is substantiated by the fact that when added to sweet cookies, the LE loses its MMP-9 inhibitory activity (Figure 5b). Regardless, the results here clearly show that for deflamin, the biochemical composition of the food matrix is crucial for its optimal function.

### 3.5. Protein Cookie Extracts Inhibit Colon Cancer Cell Migration

**Figure 6:**
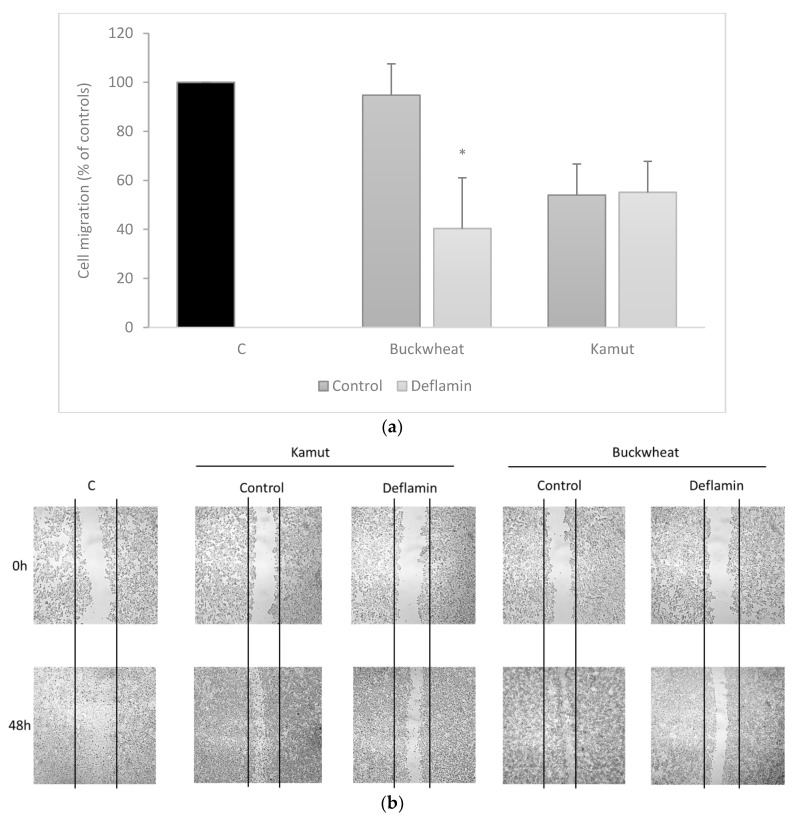
HT29 cell migration after exposure to cookies as determined by the wound healing assays: (**a**) Relative migration rates. Values are the averages of three replicate tests ± SD and are expressed as percentages of wound closure in relation to 0 h. Note: * represents *p* < 0.05 when compared to controls. (**b**) Representative images of cell migration demonstrating the inhibitory effects of kamut and buckwheat cookies. Cells were exposed to 100 µg protein.mL^−1^.

Since our previous studies showed that the LE had an ability to reduce colon cancer cell migration [8,9], we further set out to determine the effects that savoury cookies might have on cancer cells. Figure 6 shows cell migration patterns of HT29 cells after 48 h exposure to the cookie protein extracts, showing the the very significant inhibitory activity of MMP-9 using the wound healing assay.

The results clearly demonstrate that the buckwheat cookie with incorporation of the lupin extract had a significant inhibitory effect on cell migration (*p* < 0.05), whereas in controls it did not, corroborating that fact that the LE maintained its activities. As for kamut cookies, both controls and LE-containing cookies reduced HT29 cell migration, suggesting that this was due to other components of this flour. Previous reports have shown that kamut can cause less inflammatory responses than wheat [15]; however, this is, to our knowledge, the first report on this type of activity in this cereal.

Overall, in the present study only the buckwheat flour demonstrated no significant effects upon the MMP inhibitor (MMPI) activity of our lupin extract, whilst maintaining its anti-inflammatory and anticancer potential. Buckwheat flour is considered a functional food and it is presumed that its proteins are responsible for health benefits, with Giménez-Bastida et al. [39] showing that buckwheat bread reduced the effects of TNF-α on migration and cell cycle in myofibroblasts, exhibiting anti-inflammatory activity [39]. Overall, the above results allow us to infer that LE-containing cookies could have great potential as functional foods.

## 4. Conclusions

Cookies are important ready-to-eat baked snacks that are consumed globally. Regarding today’s market demands, cookies are becoming increasingly popular as functional foods with the use of alternative flours, providing both added nutritional value and bioactivity. In the present study, an efficient delivery method for incorporating a bioactive lupin extract into cookies using different alternative flours was presented. Technologically, it was found that the incorporation of the lupin extract has an impact on the characteristics of the dough and the final product, depending on the type of flour. The biochemical compositions of all cookies with lupin extract presented higher protein and ash contents when compared to the control cookies. The only flour that did not interfere with the desired nutraceutical activities was buckwheat, because it showed higher bioactivity against MMP-9 activity whilst maintaining strong inhibition of colon cancer migration. Furthermore, lupin-enriched buckwheat cookies showed improved colour, rendering their appearance more attractive to consumers; hence, buckwheat cookies were demonstrated to be a good vehicle to deliver a potent nutraceutical from lupin in preventive and curative diets, particularly in diets used to treat inflammation and cancer diseases of the gastrointestinal tract. In the near future, sensory analysis of the buckwheat cookies should be carried out to evaluate the acceptability of this new product by consumers.

## 5. Patents

DEFLAMIN: Therapeutic protein. PCT International Patent Application No. PCT/EP2017/075020. Filed on 30 September 2017. Available at: https://patentscope.wipo.int/search/en/detail.jsf?docId=WO2018060528&recNum=4&office=&queryString=FP%3A%28075020%29&prevFilter=&sortOption=Pub+Date+Desc&maxRec=48.

## Figures and Tables

**Table 1:** The ΔE*, L*, a*, b* and water activity (a_w_) values for control and LE cookies. Values are the averages of at least five experiments ± SD, except ΔE*, which is the difference between control and LE cookies. Note: * represents *p* < 0.05 and ** represents *p* < 0.001 when compared with the corresponding control cookie.

		Buckwheat	Rice	Oat	Kamut	Spelt
ΔE*		5.98	17.13	5.20	2.88	14.64
L*	Control	63.71 ± 1.94	78.63 ± 1.30	66.33 ± 2.72	68.93 ± 1.33	81.82 ± 2.08
	Lupin	60.75 ± 2.8 *	71.04 ± 1.99 **	65.76 ± 2.03	68.13 ± 1.96	71.86 ± 5.93 **
a*	Control	7.70 ± 0.65	−1.04 ± 0.09	4.20 ± 0.49	5.64 ± 0.62	2.13 ± 0.36
	Lupin	5.51 ± 0.53 **	4.01 ± 0.55 **	6.53 ± 0.54 **	8.75 ± 0.97 **	9.45 ± 0.79 **
b*	Control	26.08 ± 2.62	16.66 ± 1.93	25.94 ± 0.46	33.10 ± 2.04	28.15 ± 2.72
	Lupin	25.25 ± 1.22	29.36 ± 1.67 **	30.21 ± 2.13 **	34.25 ± 2.77	34.38 ± 1.28 **
a_w_	Control	0.09 ± 0.01	0.19 ± 0.01	0.07 ± 0.00	0.53 ± 0.01	0.57 ± 0.01
	Lupin	0.54 ± 0.02 **	0.38 ± 0.00 **	0.28 ± 0.01 **	0.67 ± 0.00 **	0.48 ± 0.00 **

**Table 2:** Chemical compositions of five different flours with and without LE (%, dry weight). Results are expressed as averages ± standard deviation (n = 3). Note: * represents *p* < 0.05 and ** represents *p* < 0.001 when comparing LE cookies with the respective controls.

		Total Ash (%)	Crude Fat (%)	Crude Protein (%)	Moisture (%)	Carbohydrates # (%)
Buckwheat	Control	4.15 ± 0.10	12.32 ± 0.06	10.14 ± 0.06	3.22 ± 0.05	70.18
	Lupin	4.78 ± 0.04 **	10.89 ± 0.03 **	13.85 ± 0.16 **	3.99 ± 0.12 **	67.75
Rice	Control	3.16 ± 0.13	7.45 ± 0.13	5.22 ± 0.06	6.65 ± 0.07	77.52
	Lupin	3.81 ± 0.10 *	9.18 ± 0.26 **	10.18 ± 0.25 **	6.80 ± 0.11	70.03
Oat	Control	4.14 ± 0.18	14.45 ± 0.13	8.03 ± 0.06	6.22 ± 0.21	67.16
	Lupin	5.15 ± 0.09 **	14.02 ± 0.04 *	12.42 ± 0.06 **	4.27 ± 0.20 **	64.15
Kamut	Control	3.48 ± 0.13	9.96 ± 0.22	10.99 ± 0.09	10.06 ± 0.11	65.51
	Lupin	4.31 ± 0.26 *	9.66 ± 0.12	14.53 ± 0.31 **	5.29 ± 0.08 **	66.21
Spelt	Control	3.42 ± 0.11	10.81 ± 0.06	8.60 ± 0.25	7.19 ± 0.05	69.98
	Lupin	4.54 ± 0.10 **	11.74 ± 0.12 **	12.60 ± 0.31 **	4.60 ± 0.23 **	66.52

# Carbohydrates were determined by differences in the mean ash, fat, protein and moisture contents.

## Data Availability

The datasets generated for this study are available on request to the corresponding author.
